# Recent advances in understanding grasslands

**DOI:** 10.12688/f1000research.15050.1

**Published:** 2018-08-30

**Authors:** Carly J. Stevens

**Affiliations:** 1 Lancaster Environment Centre, Lancaster University, Lancaster, UK

**Keywords:** Climate change, diversity, ecosystem services, ecosystem stability, grassland ecology, invasive species, nutrient addition, plant-soil interactions.

## Abstract

Grasslands are a vitally important ecosystem, supporting a wide range of ecosystem services and high levels of biodiversity. As a consequence, they have long been a focus for ecologists, playing host to some of the world’s longest-running ecological experiments and providing the inspiration for many long-standing theories and debates. Because the field of grassland ecology is broad, encompassing many areas of ecology, this article picks some areas of particular debate and development to look at recent advances. The areas include relationships between diversity and productivity, ecosystem stability and ecosystem service provision, global change threats from nutrient addition, invasive species, climate change, and plant soil interactions.

## Introduction

Grasslands cover 40% of the world’s terrestrial surface ^1^, and they are found on all continents except Antarctica, in a wide range of climates, and on a wide range of soil types. They can vary in their species richness from monocultures up to botanically species-rich habitats which support diverse animal communities. Grasslands are typically dominated by grasses ( *Poaceae*) and other grass-like plants. Some grasslands occur naturally, whilst others must be maintained by active management such as cutting or grazing. They are also incredibly important to mankind, providing many different services. Unsurprisingly, given their importance, extent, and variation, grasslands have been a focus for many ecologists and the home of many ecological theories, some of which remain intensely debated. In this article, I will highlight some of the recent advances in understanding grasslands. The field of grassland ecology is broad and developing rapidly. A Web of Science search with grasslands as a key word reveals almost 9,000 papers published between January 2016 and May 2018. As a consequence, this is not an exhaustive review but rather focusses on some key causes and consequences of biodiversity declines in grasslands, picking out some areas of key developments, controversies, and findings that I believe have helped us to advance our understanding of how these complex, intriguing, and beautiful ecosystems work.

## Variation in diversity between grasslands

### Productivity–diversity relationships

Grasslands across the world vary hugely in both physical and biological characteristics, and explaining relationships between them has led to much discussion. One of the fiercest ecological debates of recent decades has concerned the relationship between productivity and diversity. The humpbacked model ^2^ predicts that in extreme environments only a few stress-tolerant species survive and diversity is low. Both productivity and diversity increase until, when resource levels are high, diversity declines again, most likely due to competition or species pools. Grasslands account for as much as one-third of the net primary production on land ^3^, and many of the original studies exploring the relationship between productivity and diversity originated in grasslands ^2, 4^. In 2011, Adler *et al*. ^5^ published a paper in *Science* that reignited the debate. Using a global dataset of 48 grasslands on five continents that were part of the Nutrient Network (NutNet), they found no consistent relationship between productivity and richness. Since then, we have seen publications that fall on both sides of the debate. Recently, Fraser *et al*. ^6^ collected data from 30 sites on six continents and performed a similar analysis to that employed by Adler *et al*. ^5^. They found strong support for the humpbacked model, with 19 of 28 sites showing significant concave-down quadratic relationships between plant species richness and productivity ^6^. They identified a number of hypotheses for why they found much stronger support for the humpbacked model than did Adler *et al*. Whilst the debate regarding whether there is a single hypothesis that can explain the relationship between plant diversity and productivity will likely rage for a long time, most authors can agree that there is a need to develop our understanding of the mechanisms that underlie the relationships identified. Grace *et al*. ^7^ recently made progress toward this objective by showing that integrative modelling, considering multiple potential drivers of both richness and diversity, has substantially higher explanatory power than bivariate analyses, arguing for more integration of ideas and simultaneous tests of their combined implications.

## Threats to grasslands

### Land-use change

Globally, land-use change is a major driver of community change and habitat loss. The impacts of land-use change can be seen across trophic groups. A recent paper has taken a functional trait approach to examine shifts in invertebrate communities in response to land-use intensification. Working across 124 grasslands of differing intensities of land-use in Germany, Simons *et al*. ^8^ collected data on a range of traits in insect and spider species to demonstrate that higher-intensity landscapes favoured smaller, more mobile, and less-specialised species. The collection of functional trait data for invertebrates is labour intensive, but the authors argue it is essential for understanding the impacts of land-use change on invertebrates.

### Response to nutrient additions

One of the most challenging issues we currently face is the extent to which we have perturbed nutrient cycles and the impact this is having on the environment. High levels of nutrient addition have long been recognised to reduce species richness. We expect that nutrient enrichment results in a switch from belowground competition for nutrients to aboveground competition for light. Because some plants are taller, they receive more light per unit size than do smaller plants, thus precipitating competitive exclusion. Up to now, there has been limited evidence to conclusively demonstrate this mechanism, but a recent study demonstrated that an increase in light asymmetry is the main cause of species loss under nutrient enrichment ^9^. DeMalach *et al*. ^9^ used a combination of light measurements through the grassland canopy and plant height in fertilised and unfertilised grasslands to calculate light asymmetry and determine the competitive effect, demonstrating that it is an increase in the rate of light decay through the canopy rather than an increase in canopy height that is responsible for the competitive effect of grasses on forbs.

Another recent advance has been in the increased recognition of multiple factors limiting production in grasslands. Two recent papers from the NutNet ^10– 12^ have demonstrated that, contrary to popular opinion, where nitrogen (or nitrogen and phosphorus) is deemed a key determinant of aboveground net primary productivity, other nutrients are important in determining production, and not only were many grasslands limited by multiple nutrients ^10^ but the number of added nutrients predicted diversity loss. Adding nutrients reduced niche dimensionality, increased productivity, and increased compositional turnover ^11^. Nutrient addition is clearly a considerable threat to grassland biodiversity, yet in many parts of the world it does not receive sufficient attention in policy or research, meaning there are many knowledge gaps we need to address.

### Invasive species

There are many different mechanisms that have been identified for the success and spread of invasive species in grasslands and other habitats. A recent paper by Broadbent *et al*. ^13^ highlighted the potential importance of root competition in the interactions between invasive and native grasses in New Zealand. This small-scale study only investigated the relationship between three species but demonstrated the importance of belowground competition, a mechanism that has received very little attention, in driving their interactions and highlights this as an area in need of future research. In contrast, a large meta-analysis was performed by Liu *et al*. ^14^ to test whether invasive species benefit more from global environmental change than do native species. They compiled a database of published studies that gave performance measures for 74 invasive plant species and 117 native plant species in response to global change drivers. They found that invasive species responded more strongly to carbon dioxide enrichment and elevated temperature (and did not respond significantly to nitrogen deposition and increased precipitation), indicating that the problems caused by invasive plant species are likely to get worse under a changing climate. This study suggested that drought may be detrimental for invasive species, as did a seedbank study in Californian annual grasslands, which found that seeds of exotic annual grasses declined whilst native annual forbs increased ^15^. Invasive species have very large impacts on grassland communities in some parts of the world, meaning that understanding and predicting these impacts is a priority.

### Climate change

Long-term experiments are critically important in ecology ^16^, and as grassland ecologists we are lucky to host some of the longest-running experiments in the world, including the 150-year-old Park Grass Experiment ^17^. For climate change research, long-term experiments are especially valuable, as it takes time for plant species to respond. One of the longest-running climate change experiments is the Buxton Climate Change Impacts Laboratory (BCCIL) in northern England ^18^. Recent research at BCCIL has demonstrated that climate change (warming and rainfall manipulation) has rapid direct impacts on the soil microbial community but also indirect impacts mediated by changes in plant species composition, which occur over longer time scales ^19^.

Shifts in species composition are likely as a result of climate change but do not necessarily result in changes to ecosystem stability ^20^. Also, using a long-term investigation, Reich *et al*. ^21^ showed that whilst in the first 12 years of carbon dioxide enrichment C _3_ plant biomass increased markedly, C _4_ plant biomass did not. This is expected, since C _4_ plants are thought to be less limited by carbon dioxide; however, in the latter 8 years of the experiment, the responses switched, with biomass depressed in C _3_ plants but not in C4 plants. Fay *et al*. ^22^ demonstrated that carbon dioxide concentrations have a strong effect on flowering in four of the five grassland species they investigated. They utilised contrasting soil conditions to demonstrate that impacts were mediated by productivity and nutrient status. The impacts of climate change will be felt globally, but we are only just beginning to understand likely impacts. In the future, we need more research to understand not only likely impacts of climate change but also how climate change and other global change drivers are likely to interact; long-term experiments will be key to achieving this.

## Effects of biodiversity loss

### Diversity–stability relationships

One of the main arguments for biodiversity conservation is that more-diverse communities will be more stable and better able to resist perturbation. A number of mechanisms have been suggested for this relationship between diversity and stability, including asynchrony (productivity of one species increases, compensating for declines in another species), portfolio effects (statistical averaging of fluctuations in species properties), and functional redundancy (species loss is compensated by other species fulfilling a similar function). A recent study of 2,671 species from 300 plots, across three regions in Germany, indicated that asynchrony was the primary driver of stability rather than diversity alone ^23^. Evidence for this is mixed, but several recent papers, including two synthesis studies combining results from multiple experiments in grasslands, have provided strong evidence in support of the argument that diversity begets stability. Hautier *et al*. ^24^ used results from 12 multiyear experiments to show that changes in biodiversity caused by a range of environmental change drivers were a major factor in determining the impact on stability, whilst Isbell *et al*. ^25^ used data from 46 experiments that manipulated grassland diversity to show that diversity increased resistance to climate events. Further results from the NutNet collaborative experiment have shown that eutrophication weakens the relationship between diversity and stability. We would expect this to occur as a result of diversity losses, but in this case it was actually due to an increase in temporal variability of productivity ^26^.

## Diversity–service provision relationships

Another often-cited negative consequence of the loss of biodiversity is that more-diverse systems support more ecosystem services. Many of the early papers in this field were conducted in grasslands, and now there is a move towards testing these relationships in the “real-world” in natural or managed grasslands. Allan *et al*. ^27^ provided strong evidence to support this, using 150 grassland plots spread across three regions of Germany as part of the Biodiversity Exploratories experiment. Their results show that diversity loss and functional composition change, caused by land-use intensification, is just as important as the land-use intensification itself in terms of its impact on ecosystem service delivery. It is not just richness that is important though: Winfree *et al*. ^28^ compared composition, richness, and abundance of pollinators to determine their relative contributions to pollination services and found that the abundance of dominant species was the most important factor whereas richness was relatively unimportant because most of the species were not responsible for service delivery. Whilst this is interesting evidence in favour of considering the composition of communities, this study was focussed on one trophic group and one ecosystem service; other recent studies have demonstrated the need for multiple trophic groups to be considered. Results also from the Biodiversity Exploratories project demonstrated that high richness in multiple trophic groups had stronger positive effects on ecosystem service provision than did richness in any single group. This was particularly true for cultural and regulating services ^29^ and for the provision of multiple ecosystem services ^30^.

## Conclusions

Grassland ecology, and the broader field of ecology, are rapidly moving fields. Developing analytical and statistical techniques combined with innovative approaches and co-ordinated networks are allowing us to address questions which we were not previously able to. However, many questions remain, and there are rarely, if ever, definitive answers to questions in ecology. Luckily, in much of grassland ecology, there is a willingness to embrace change and accept that rules are there to be broken. There are, however, unprecedented threats to grassland habitats through climate change, nutrient deposition, invasive species, and habitat loss, to name a few, so the need to understand impacts and protect valuable habitats is more pressing than ever.

## Abbreviations

BCCIL, Buxton Climate Change Impacts Laboratory; NutNet, Nutrient Network
